# Project nature: promoting outdoor physical activity in children via primary care

**DOI:** 10.1186/s12875-024-02297-5

**Published:** 2024-02-23

**Authors:** Georgia M. Griffin, Carolina Nieto, Kirsten Senturia, Marshall Brown, Kimberly Garrett, Elizabeth Nguyen, Danette Glassy, Emily Kroshus, Pooja Tandon

**Affiliations:** 1 Department of Pediatrics, University of Washington, 4800 Sandpoint Way NE, Seattle, WA 98105 USA; 2 Seattle Children’s Research Institute, 1920 Terry Ave, Seattle, WA 98101 USA; 3 University of Washington School of Public Health, 3980 15th Ave NE, Seattle, WA 98195 USA; 4 Department of Primary Care & Rural Medicine, Texas A&M School of Medicine, 2900 E 29th Street, Suite 100, Bryan, TX 77802 USA; 5 BestStart Washington, PO Box 318, Mercer Island, WA 98040 USA

**Keywords:** Nature contact, Outdoor play, Physical activity, Well-child care, Primary care, Health equity

## Abstract

**Background:**

Families face a range of barriers in supporting their children’s active play in nature including family circumstances, environmental constraints, and behavioral factors. Evidence-based strategies to address these barriers are needed. We aimed to develop and pilot test a primary care-based family-centered behavioral intervention to promote active outdoor play in 4–10 year-old children.

**Methods:**

Project Nature, a provider-delivered intervention that provides informational resources and an age-appropriate toy for nature play, was initially developed for children ages 0–3. With stakeholder input, we adapted existing materials for 4–10 year-olds and conducted usability testing at an urban clinic serving families from diverse backgrounds. Subsequently, we conducted a mix-methods pilot study to evaluate intervention feasibility and acceptability. Parents of 4–10 year-olds completed pre- and post-surveys (*n* = 22), and a purposive subset (*n* = 10) completed qualitative interviews. Post-intervention, pediatric providers (*n* = 4) were interviewed about their implementation experiences.

**Results:**

The majority (82%) of parents liked the information provided and the remaining (18%) were neutral. Qualitatively, parents reported that: the toy provided a tangible element to help children and parents be active, they did not use the website, and they wished the intervention emphasized strategies for physical activity during cold and wet seasons. Providers felt the materials facilitated discussion about behavior change with families. There were no statistically significant changes in PA and outdoor time pre- and post-intervention.

**Conclusions:**

Project Nature was welcomed by providers and families and may be a practical intervention to promote outdoor active play during well-child visits. Providing an age-appropriate nature toy seemed to be a critical component of the intervention, and may be worth the additional cost, time and storage space required by clinics. Building from these results, Project Nature should be revised to better support active outdoor play during suboptimal weather and evaluated to test its efficacy in a fully-powered trial.

**Supplementary Information:**

The online version contains supplementary material available at 10.1186/s12875-024-02297-5.

## Background

The health benefits of daily physical activity (PA) and time outdoors are well-recognized [1–3]. For children, time outdoors is strongly associated with increased PA [1] and a range of other benefits including improved mental health, healthier weight status, and less myopia [3, 4]. The American Academy of Pediatrics and other experts recommend children and adolescents play outdoors daily and participate in moderate-to-vigorous PA (MVPA) 60 minutes/day to improve physical, mental, and cognitive health [5–8]. In 2019 only 23% of U.S. youth met these recommendations, a percentage that has been declining over time [9] and especially since the COVID-19 pandemic [10]. A growing body of evidence indicates that nature contact can confer both physical and mental health benefits for children of all ages. A recent systematic review of almost 300 studies concluded that the current literature supports a positive relationship between nature contact and children’s health, and recommended advocating for strategies that promote equitable nature contact for children in places where they live, play, and learn [3]. Parents have identified a range of barriers to supporting children’s active play in nature including family circumstances (e.g., time, finances, single parenting), environmental constraints (e.g., access to safe outdoor play spaces, transportation, finding age-appropriate activities), and behavioral factors (e.g., previous experiences in nature, safety, weather concerns) [11]. Many barriers stem from structural inequities rooted in historical, racial discriminatory practices such as “redlining” [12] that created disparities in parks access.

While addressing structural barriers to nature access is paramount to supporting equitable access to outdoor play [11, 13], there is also opportunity for family-centered interventions [11, 13]. It is vital to establish active play habits early, as PA strongly tracks from early childhood to adulthood [14]. “Prescriptions” to encourage time in nature [15–18] provide directive information about what families “should” do, but may not address context-specific barriers to outdoor play. A recent study conducted by our team found that parents of school aged children wanted information about opportunities for nature play near their homes, including how to access nature and support children’s safe play under different weather conditions [11]. Evidence-based primary care strategies to promote children’s active play in nature and address existing barriers are needed.

Project Nature (PN), a provider-delivered intervention developed initially for children ages 0–3, addresses some of these limitations using an educational brochure (that describes benefits to active play in nature and activity ideas), a website (that shares local resources including parks, green spaces, and nature programs), and an age-appropriate nature toy (that can be used for outdoor play). The development of this intervention was guided by the WHO’s Commission on Social Determinants of Health framework [19] and our formative work pointed to well-child visits as an appropriate setting to support children’s outdoor play [11]. Pediatric providers employing PN identified a need for a similar intervention aimed at older school-aged children.

The aims of this multi-phase study were to (1) conduct an adaptation needs assessment of the existing preschool age intervention, (2) adapt PN for school-aged children with parent and provider input, and (3) test the feasibility, acceptability, and preliminary efficacy of this adapted intervention.

## Methods

### Study design and personnel

**Fig. 1:**
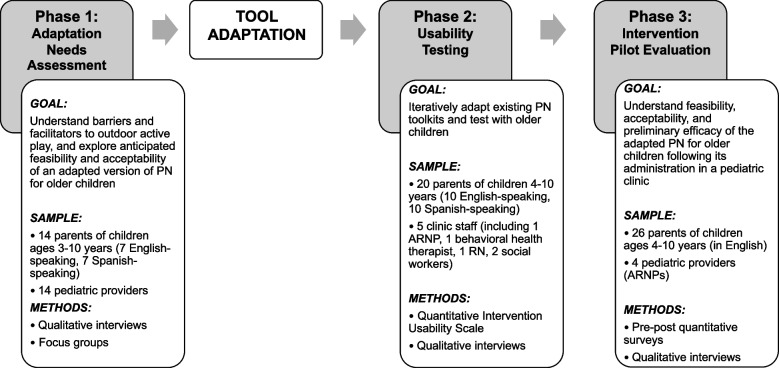
Study phases and components

This study was conducted in Seattle, WA from 2020 to 2023, and included three phases: (1) adaptation needs assessment, (2) material adaptation and usability testing, and (3) pilot evaluation. See Fig. 1 for a description with details of each component. The Seattle Children’s Hospital Institutional Review Board approved the study. This report conforms to the Standards for Reporting Qualitative Research [20]. Our multidisciplinary team included individuals with expertise in pediatrics (PT, DG), sports medicine (GG, LN), public health research (EK), anthropology and qualitative research (KS, CN), research coordination (KG), and data analysis (MB). Our team members identify across many disciplines, ethnicities, languages, and parenting experiences; these diversities and viewpoints informed study design, data collection and data analysis. We employed triangulation at many stages so data from each source contributed to our robust understanding of the phenomena. In this study we used an inclusive definition of “parent” as one of a child’s primary guardians accompanying the child to their clinic visit.

### Phase 1: adaptation needs assessment

A convenience sample of 14 (7 English-speaking and 7 Spanish-speaking) parents of children age 3–10 were recruited from pediatric clinics in Seattle that serve high proportions of patients with public insurance. We selected these clinics recognizing that outdoor play in nature is less accessible for families with resource constraints and those who face cultural barriers. 14 pediatric providers were recruited via word of mouth and a message in the Washington Chapter of the American Academy of Pediatrics newsletter. Interviews with parents centered around family barriers to outdoor play and conversations about playing in nature with their pediatric provider (see Additional file 1), and were conducted by native speakers, professionally translated and transcribed, and then spot-checked by native speakers for accuracy. Focus groups with providers explored feasibility and acceptability of adapting PN for older children (see Additional file 2).

### Phase 2: material adaptation and usability testing

Incorporating themes from the adaptation needs assessment, we systematically adapted the existing PN materials (designed for 0–3 year old children) for older children 4–10 years old. Proposed adaptations were brought to our multidisciplinary planning group for input on developmental appropriateness, consistency with the core components of PN, and considerations related to equity and inclusion. This feedback was: synthesized into an initial draft of a brochure, revised by a graphic designer, and reviewed by our full team.

Next, we conducted usability testing with the adapted intervention with a sample of English- and Spanish-speaking parents and clinic staff at one pediatric health clinic serving primarily publicly insured children in an economically and racially diverse neighborhood in Seattle. Spanish interviews were conducted by native speakers. A convenience sample of 20 (10 English-speaking and 10 Spanish-speaking) parents of 4–10 year-old children seeking care were invited to review PN materials and provide mixed method feedback on their acceptability and usability. 5 staff members were interviewed (1 ARNP, 1 behavioral health therapist, 1 RN, 2 social workers). All participants verbally provided informed consent. We measured usability with Lyon et al.’s Usability Evaluation for Evidence-Based Psychosocial Interventions scale [21]. Qualitative interviews were conducted with each of the participants. Interview guides can be found in the Additional file 3. Feedback was synthesized in the moment following each interview and taken back to the team. The results were used to finalize the age-adapted PN intervention components.

### Phase 3: pilot evaluation

#### Sample and procedure

We held a meeting with interested providers at our partner clinic and provided a written guide for how providers can introduce and discuss PN with families. Providers could tailor conversations based on families’ circumstances, needs and barriers. Printed brochures and nature toys were delivered to the clinic.

We recruited families with the following eligibility criteria were: (1) child scheduled for a 4–10 year-old well-child visit with a participating healthcare provider, (2) parent spoke English, and (3) verbal consent obtained from a parent or guardian prior to the visit in person or via phone call. Parents were asked to complete quantitative surveys via email, phone call, or paper before the scheduled well-child visit. Three weeks post-intervention, parents were asked to complete a post-intervention survey. Surveyed parents were subsequently invited to complete qualitative interviews. After reviewing our initial 10 transcripts, we ascertained that data sufficiency had been achieved and data collection was complete. Post-intervention, we held phone interviews with participating providers (4 ARNPs) regarding clinic feasibility and impressions of the intervention.

#### Survey measures

Pre- and post-survey topics included: MVPA participation by child and parent independently for at least 20 minutes/day; co-participation in PA and outdoor time; and prior discussions with the child’s healthcare provider about PA and outdoor time. Although the recommendation for children 6 and older is 60 minutes/day of MVPA and 150 minutes/week for adults, the guidance for younger children is less concrete, so we chose the 20 minute/day mark to be able to capture some PA in an increment that may be more reasonably impacted by the PN intervention. The survey also inquired about barriers that may prevent the child from being physically active and demographics. The post-survey assessed perceptions of the intervention’s feasibility and accessibility using Weiner et al.’s Acceptability of Intervention Measure, a validated 4-question self-report scale [22], as well as self-reported implementation of toy and brochure. The survey can be found in Additional file 4.

#### Qualitative measures

Using a phenomenological approach, our qualitative interview guides were developed according to study goals and adjusted as necessary per standard qualitative methodology [23, 24]. Individual interviews with parents focused on acceptability, feasibility and preliminary efficacy of the intervention. Interviews with providers addressed acceptability and feasibility of the intervention in the clinic setting. Parent and provider interview guides can be found in the Additional file 5 and 6.

### Data analysis

Parent and child demographics were summarized descriptively. Mean and standard deviations were calculated for numeric outcomes pre and post intervention and compared statistically with paired t-tests. This was a Phase 1b behavioral study, and thus not powered to detect change quantitatively.

Interviews and focus groups (in Phase 1 and 3) were digitally recorded, professionally transcribed (and translated where appropriate), and spot checked by interviewers to ensure data integrity [25, 26]. In the results, quotes are identified by data set (where 1 = adaptation needs assessment, 2 = pilot evaluation), qualitative tool (IN = interview, FG = Focus group), parent versus provider (PA = Parent, PR = provider), language (EN = English, SP = Spanish) and participant number.

Data were uploaded into Dedoose Version 7.0.23 (Sociocultural Research Consultants, Los Angeles, California) for coding and thematic analysis following procedures outlined by Braun and Clarke [27]. Steps to codebook development were as follows: initial codes were derived from study goals; codes were augmented by a reading of two transcripts; codes were tested on three additional transcripts by two coders; the codebook was edited until an exhaustive but manageable code list was reached. We used a multi-step approach to developing the codebook which allowed for both deductive codes (e.g., Perceptions and Usability of PN) extracted from study goals, instruments, frameworks, and inductive codes (e.g., Weather and Child unmotivated to go outside as barriers to do PA) emerging from review of transcripts.

Transcripts were open coded by two coders (KS, CN) who were blind to each other’s coding and differences were resolved by discussion until 100% agreement was reached. During synthesis, coded excerpts were systematically summarized into themes and subthemes with associated quotes.

## Results

### Adaptation needs assessment

Demographics for the Adaptation Needs Assessment can be found in Tandon et al. (2022) [11]. Several key needs and key barriers for the adapted materials emerged from the pediatric provider focus groups and parent interviews, which are summarized in Tables 1 and 2 respectively.

**Table 1:** Key needs for the adaptation as identified by providers and parents with relevant quotes

Key Needs	Examples & Suggestions	Illustrative Quotes	
		Provider	Parent
**Engaging**	• Success depends on child and family being engaged • Toy is engaging for child	*… it kind of depends on the family because … that child – who’s 5 years old, whose BMI is not even on the chart – is just glued to his games and his parents iPhone. And I don’t think giving him this would interest him at all…. I think it also has to do with how much enthusiasm the parents bring with it. [1-FG-PR-EN-113]*	*The bug thing definitely would have been a huge hit with him. He would have wanted to go out and collect bugs all the time. [1-IN-PA-EN-15]*
**Likable**	• Toy is fun and exciting	*I can imagine something similar happening with this, it becoming something that’s exciting to look forward to at their well child visit, especially for families without as many resources to provide those things. [1-FG-PR-EN-105]*	*Getting new stuff in the mail is always exciting and those are all things for outdoors so it might just make them pick them up and run outside. … I don’t think that every family would have bug catching kit. [1-IN-PA-EN-102]*
**Tangible**	• Concrete toy/object is motivating to parents AND children	*I feel like families respond really well to getting that concrete thing, because when we’re just telling them do this or do that, I feel like sometimes it’s just another item on the long list of things that various professionals have told them they should be doing that they’re not doing. [1-FG-PR-EN-108]*	*You feel more encouraged as a parent to do things. ... Having someone say, “This is going to help your daughter with this.” “Okay, let’s go. I will do it.” Especially if you have these materials at hand. [1-IN-PA-SP-96]*
**Avenue for sharing activity ideas**	• Provide ideas for free outdoor activities & instructions • Share environmental education opportunities	*N/A*	*…different ways of doing what you could do with the shovel and the watering can or bug catcher… to generate conversation that way. [1-IN-PA-EN-101]* *…we have to instill in children that we have to take care of nature, it helps us breathe cleaner. [1-IN-PA-SP-05]*
**Formatted to be child-friendly, accessible, and inclusive**	• Inclusive visuals	*The pictures are great for kids to do the scavenger. [1-FG-PR-EN-108]* *…including kids of different racial and ethnic backgrounds, and including people in hijabs, and including people with mobility challenges or other types of disabilities – development disability,* et cetera *– I think including different types of families. [1-FG-PR-EN-108]*	N/A
**Equitable**	• Language & literacy • Sustainability	*We have a lot of parents who don’t read in any language, and so having printed materials, even if we can get different languages, is not super helpful. So, having something that’s very graphic and easy to explain. [1-FG-PR-EN-108]* *If it all of a sudden goes away that’s pretty disappointing, and so, it would be nice if it was something that we could sustain over a long period of time. [1-FG-PR-EN-105]*	*N/A*

**Table 2:** Key barriers for the of the intervention as identified by providers and parents with relevant quotes

Key Barriers	Examples & Suggestions	Illustrative Quotes	
		Provider	Parent
**Transportation & expenses**	• The tool does not address structural barriers	*It’s amazing we live in this beautiful place with amazing outdoor resources, but … transportation can be a barrier, having to get somewhere, having to pay for parking … having things that are very neighborhood-based that are pointing out the ways in which nature is available in small doses, even in your apartment complex, and ways to be able to encourage physical activity with things that are best done outdoors. [1-FG-PR-EN-105]*	*I’m just concerned about other families. What if they do have no transportation to go out?....Would there be some type of assistance for them so that they can go out? Just any type of assistance, or financial assistance to support them. [1-IN-PA-EN-13]*
**Neighborhood context and safety**	• The tool does not address structural barriers	*…how close are they to this source and how safe is it for them to – or does mom drive? Can mom get them there, or a parent, or whoever? And how safe is it for them to walk if nobody can drive them there? [1-FG-PR-EN-113]*	*it has a space to do the activities that need to be done, as I live in an apartment and do not have a balcony and I would have to go out to the hallway and it does not stop being complicated because they do not let plant any flowers, nor dig. [1-IN-PA-SP-05]*
**Children preferring sedentary, indoor activities**	• Fun activities may overcome children’s desire to be indoors and sedentary	*If they make this into a big, special activity or something fun, and make it more exciting and appealing than their iPads, and iPhones, and videogames, then I think it could totally work. [1-FG-PR-EN-113]*	*I would say, things that will persuade kids to go out more. I don’t necessarily have anything in mind. But definitely not like, eat junk food or something that will encourage them to stay indoors, right? [1-IN-PA-EN-00]*
**Parent energy, time, absence**	• Toolkit should be easy to use	*I’m not sure if a lot of parents would be able to put in all that enthusiasm, especially if they’re already having all these other barriers and if they’re tired or if they don’t have time. Things like that.* *[1-FG-PR-EN-113]*	*I think maybe only things that would get in the way would be the same issues of we’re too tired. I think that is probably the biggest issue. …. I think it’s mostly us just being exhausted. [1-IN-PA-EN-15]*
**Winter weather**	• Rain gear may mitigate this barrier • Toys and activities should be suitable for winter weather	*The concept of having rain gear, I imagine there’s some basic awareness of that, but the parents themselves have never seen this. [1-FG-PR-EN-101]*	*…even a little bucket they could dig in our front yard. The weather though it’s cold. It’s cold and the dirt’s cold and they don’t want to put their little hands in the cold dirt. So, weather. [1-IN-PA-EN-102]*
**Clinic feasibility**	• Storage space • Clinic culture/staffing • Visit duration	*I don’t think it would be something that I, personally, or any of the other providers in my clinic would want to take on as a responsibility of making sure we have an inventory of this. [1-FG-PR-EN-108]*	*N/A*

Providers and parents emphasized the importance of the intervention being engaging, fun, and exciting. Both felt that concrete physical materials such as a toy and brochure can be particularly motivating. Images representing families from many different backgrounds would make the materials child-friendly and inclusive. It would also be important to consider family literacy and language barriers. There were concerns that it would be difficult for the intervention to address structural barriers to active play outside including transportation, expenses, and neighborhood safety. Other barriers, such as children preferring sedentary, indoor activities and suboptimal weather could potentially be addressed by the intervention.

Providers identified possible clinic-centered challenges to PN implementation including inadequate storage space, established clinic culture and habits inhibiting distribution and inventory of PN materials, and the limited length of well-child visits.

### Material adaptation & usability testing

Demographics for the Material Adaptation & Usability Testing are displayed in Table 3. The key needs highlighted by the needs assessment were used to adapt the core components of PN for 4–10 year-olds and to diverse families. Table 4 summarizes iterative feedback from parents and clinic staff on the written materials and nature toy, which was incorporated into the final adapted materials. Parents wished for a varied selection of nature toys to match their children’s diverse interests; the research team used parents’ suggestions to finalize the following nature toy options: kite, jump rope, bubble wand, frisbee, colored chalk, bug catcher and magnifying glass, and shovel and seeds. When surveyed, parents and clinic staff who participated in usability testing described PN as appealing to use often, easy to understand, and easy to learn how to use.

**Table 3:** Material adaptation & usability testing parent demographics

***Parent/guardian characteristic***	***N* = 10**^**1**^
*Age*	
20–25 years	3 (30%)
30–39 years	6 (60%)
40–49 years	1 (10%)
*Gender*	
Male	3 (30%)
Female	7 (70%)
*Number of children between the ages of 3–10 years*	
1	5 (50%)
2	3 (30%)
3	2 (20%)
*Ethnicity*	
Mexican, Hispanic, or Latin American descent	3 (30%)
*Race*	
Asian or Pacific Islander	3 (30%)
Black or African American	3 (30%)
Hispanic or Latino	2 (20%)
Native American or Alaska Native	0 (0%)
White or Caucasian	1 (10%)
Multiracial or Biracial	1 (10%)
***Clinic staff characteristic***	***N*** **= 5**^**1**^
*Age*	
*30–39 years*	2 (40%)
*40–49 years*	3 (60%)
*Gender*	
Male	1 (20%)
Female	4 (80%)
*Ethnicity*	
Hispanic, Latin American, or Mexican descent	1 (20%)
*Race*	
Asian or Pacific Islander	1 (10%)
Black or African American	0 (0%)
Hispanic or Latino	0 (0%)
Native American or Alaska Native	0 (0%)
White or Caucasian	4 (80%)
Multiracial or Biracial	0 (0%)
*Years in practice*	
0–5	2 (40%)
6–10	1 (20%)
11–15	1 (20%)
16–20	1 (20%)
^1^n (%)

**Table 4:** Qualitative usability testing and PN adaptation

Core Tool Component	Parent Input	Clinic Staff Input	Adaptation
**Written materials**	• Should include instructions on how to use the nature toys • Should include additional outdoor activity ideas and community resources (e.g. information about local parks or library scavenger hunts) • Should describe the specific benefits of PA • Pictures are great, but should include representation of children with darker skin tones and pictures of families in different living environments • Should be child-friendly and child-oriented (e.g. encourage them to play outside instead of use technology)	• Should include pictures of children from different racial and ethnic backgrounds and with different disabilities	• Brochure includes pictures of diverse families • Brochure describes the health benefits of active play outdoors and ideas for activities in ways children can understand • Brochure and website emphasize activities that families can do together
**Nature toy**	• Parents had varied opinions on which toy their child would prefer • Should match children’s varied interests • Should be age appropriate	• Should be not too large to store	• Offer families a choice in toy • Toy options selected: kite; jump rope; bubble wand; frisbee; colored chalk; bug catcher and magnifying glass; shovel and seeds

### Pilot evaluation

Demographics of participants who completed pre- and post-surveys are displayed in Table 5. 26 families received the intervention and 22 parents completed both pre- and post-intervention surveys. Of those 22, 10 completed qualitative interviews. Of the 6 pediatric providers who participated in the pilot, we interviewed 4 ARNPs.

**Table 5:** Pilot evaluation parent and child demographics

*Participant characteristic*	*N* = 22^1^
**Parents**	
*Parent gender identity*	
Man	2 (9.1%)
Woman	20 (91%)
*Parent ethnicity*	
Hispanic, Latino, or of Spanish origin	2 (9.1%)
*Parent race*	
Asian	5 (23%)
Black or African American	11 (50%)
Middle Eastern or North African	0 (0%)
Multiracial or mixed race	0 (0%)
Native American and/or Alaska Native	0 (0%)
Native Hawaiian and/or Pacific Islander	0 (0%)
White	4 (18%)
None of the above or chose not to answer this question	2 (9%)
*Parent age* (*N* = 21)	
Year, Mean (SD) [Median (Range)]	38.8 (6.3) [39 (29–54)]
**Children**	
*Age of child attending the Well Child Checkup*	
4	4 (20%)
5	6 (30%)
6	5 (25%)
7	2 (10%)
8	1 (5.0%)
9	1 (5.0%)
10	1 (5.0%)
Age missing	2
*Child gender identity*	
Boy	11 (50%)
Girl	11 (50%)
*Child ethnicity*	
Hispanic, Latino, or of Spanish origin	2 (9.5%)
Non-Hispanic, Latino, or of Spanish origin	19 (90%)
None of the above or chose not to answer this question	1 (4.5%)
*Child race*	
Asian	5 (23%)
Black or African American	10 (45%)
Middle Eastern or North African	0 (0%)
Multiracial or mixed race	1 (4.5%)
Native American and/or Alaska Native	0 (0%)
Native Hawaiian and/or /Pacific Islander	0 (0%)
White	7 (32%)
None of the above or chose not to answer this question	1 (4.5%)
*Child has mental health condition (*i.e. *anxiety, depression)*	
Yes	0 (0%)
*Child has behavioral or neurodevelopmental condition (*i.e. *ADHD, autism, learning disability)*	
Yes	2 (9.1%)
*Child has chronic physical health condition (*i.e. *asthma, diabetes, inflammatory bowel disease)*	
Yes	2 (9.1%)
^1^n (%)	

Here we report our findings from the Pilot Evaluation organized by 1) feasibility and acceptability, and 2) preliminary efficacy. Qualitative feedback from providers and parents on their experience with the intervention is summarized in Table 6.

**Table 6:** Relevant quotes by domain, subdomain, data source, respondent

Domain	Subdomains & Comments	Provider Quotes	Parent Quotes
**Feasibility**	**Clinic-centered challenges:** • Storage • Clinic culture & staffing • Visit duration	*It doesn’t take long to give a toy out and to explain like part of the project and give them the brochure. I didn’t necessarily read the whole brochure to them or anything, but I was available for any questions…. [2-IN-PR-EN-03]*	*N/A*
	**Patient-centered challenges:** • Language/ literacy • Sustainability	*The only downside I could think of is if it’s not in a language that the patient can read or if the family can read, then it’s not really beneficial for them. …. [2-IN-PR-EN-04]*	*One time maybe a few days later, I reminded him we had it, and he picked it up and took it, and went outside, but it didn’t last very long. [2-IN-PA-EN-03]* *Seven days straight. And then he gave up on it..[2-IN-PA-EN-08]*
**Acceptability**	**TOY** **Likable aspects:** • Fun & exciting • Portable & storable **Disappointing aspects:** • Quality • Durability	*Oh, they loved the toy. And they gave them the option of like what toys they wanted. Yeah, most of them were really, really happy with the choices that were allowed. [2-IN-PR-EN-04]*	*I always thought it was lightweight and small, and I could pack it somewhere. [2-IN-PA-EN-02]* *… [My son] was really excited to get bugs in this jar and look at them. And then [my daughter] was excited to see her sunflower seeds grow. [2-IN-PA-EN-05]* *I know it’s hard because you’re working with a budget, but I think having better quality or more durable toys would see better results and actually changing behavior. [2-IN-PA-EN-03]*
	**BROCHURE** **Helpful aspects:** • Child-friendly • Accessible formatting **Suggestions for improvement:** • Include ideas for cold/wet weather • Consider formatting as a calendar	*… it was helpful in framing conversation and I think that it’s helpful for parents to have something in their hands to remember what we talked about …. But I felt like overall, it was pretty easy to read and interact with. [2-IN-PR-EN-01]*	*It’s very easy to, like, have ideas to do things outside when the weather’s nice, but giving ideas of what to do when the weather is not so good. [2 IN-PA-EN-02]* *If there was something like … here’s a …calendar of like -- here’s the activities. Mark off, like on the calendar, what was done. Kind of the way the libraries do, like the reading calendars over the summer. [2-IN-PA-EN-09]*
	**WEBSITE:** • Parents unaware of website • Reliant on providers to advertise	N/A	*I don’t know if it was emphasized enough that there was another tool to go along with the brochure and the participation toy with the study. Maybe emphasize it a little more. [2-IN-PA-EN-06]*
**Barriers to using PN**	**Transportation & expense** (toolkit does not address structural barriers)	*I’m just thinking about how a good number of our families at Odessa Brown do live in apartments…it’s just not as easily accessible for them to go outside. [2-IN-PR-EN-03]*	*I think really having like resources for families who, you know, might not be able to, you know, attain the ability to attend these kinds of activities. Us, you know, as working parents do have to go to work and provide for a family. [2-IN-PA-EN-06]*
	**Neighborhood context & safety** (toolkit does not address structural barriers)	*They’d have to go down the stairs, you know, like find a safe place. And so I think depending on where they actually live, it will affect how frequently they use like their outdoor tool, I guess, or toy. [2-IN-PR-EN-03]*	*N/A*
	**Children tired & prefer sedentary activities**	*N/A*	*Sometimes the kids after school, they are just sort of burned out and they’re tired and they want to just have like downtime. Unfortunately, I think like TV and iPads… if you gave them a choice, they would choose that over going outside sometimes. But once they get outside, they’re always happy to do that. [2-IN-PA-EN-09]*
	**Parent energy, time, & absence**	*N/A*	*I work overtime because my shift is like from 7:00 to 3:30, but sometimes I work until 5:00. In that case, we don’t have time for other activities. She has to go to back sleep at 7:00. So, we only have like an hour or two to do her homework. [2-IN-PA-EN-09]*
	**Weather** (summer toys not applicable during winter)	*I think simple toys … like jump rope…are fun and good in the summer time, but tend to be a little bit messier and cold, … there’s the majority of the winter in the Seattle area. [2-IN-PR-EN-03]*	*I recall wondering, you know -- oh, what the like -- you know, it’s very easy to do stuff outside with [my daughter] when the weather’s nice, but it kind of turns super rainy. What, like, what are some ideas of what to do then? [2-IN-PA-EN-02]*
**Efficacy**	**Contributors to efficacy:** • Concrete, tangible object was motivating to parents AND children • Facilitated conversations & play • Provided ideas	*I thought that families liked the intervention. It was acceptable to them and it facilitated conversations about specific ways that kids could engage in outside play. [2-IN-PR-EN-03]*	*I liked that it gave me an idea of another fun activity to do with her outside at the park instead of just playing at the playground…. [2-IN-PA-EN-02]* *… I’m not that active as her… So that [kite] makes me run, too, because I have to start it for her to fly. So, it was kind of interesting that it really made me open my eye too. [2-IN-PA-EN-01]*
	**Limitations to efficacy:** • Dependent on compatibility • Dependent on engagement	*They really loved picking out the activities or the little kind of things that we had available to them at the clinic. And then they were pretty motivated to try to do some more things outside. [2-IN-PR-EN-04]*	*I thought it was cute, because she actually, she likes plants. She likes seeing how they come up. She likes looking at all different kinds of plants. The colors is just something that she likes doing. So, I was pretty proud of her that she picked something that she was interested in. [2-IN-PA-EN-07]*

### Feasibility and acceptability

Parents generally liked the information about PN discussed by their pediatrician, the toy, and the brochure (Table 7). The mean score on Weiner et al.’s validated 4-item Acceptability of Intervention measure was 4.3, SD = 0.8, out of a range of 1 to 5, where higher scores indicate greater acceptability. On average, participants endorsed a response of “agree” or “completely agree” to items about the acceptability of the intervention. 82% of families who were given the PN kit by their provider reported using it.

**Table 7:** Parent perceptions about components of PN

	Liked	Neutral about it	Disliked	Do not remember
**Information discussed by pediatrician**	82.1%	18%	0%	0%
**PN toy**	91%	18%	4.5%	4.5%
**PN brochure**	87%	32%	9.1%	4.5%

Interviews confirmed that PN as a whole was well-received and welcomed by both parents and providers.

With few exceptions, parents perceived the nature toys to be fun and conveniently sized (e.g. portable and storable). Parents were pleased that toys matched their children’s individual interests. Children did not show a predilection to choosing certain toys over others; the least expensive toys (e.g. chalk, bubble wand, and frisbee) were just as popular as the more expensive ones (e.g. seeds and shovel).

Some parents mentioned that their children were unmotivated to do PA, preferring screen-based and sedentary activities; the children enjoyed PA once they were outdoors, but the process of persuading them to go out could be hard. Parents liked that the PN toy reminded them to take their children outdoors for PA and provided more ideas for how to encourage their children to be active. Parents raised concerns about the toy durability; they observed that PN might not be sustainable long term because children were initially excited but lost enthusiasm over time or when toys broke.

While the brochure was well-liked by parents in survey responses, the majority of parents interviewed did not remember using the brochure. Parents wished that the brochure included more ideas for activities to do when the weather is less favorable for outdoor play. Parents suggested changing the format (e.g. to activity book or calendar) and including more activities ideas besides those involving the PN toy (e.g. scavenger hunt).

Most parents were not aware of the website and therefore did not visit it. However, parents did express interest in visiting the website if they had known about it and suggested that providers should emphasize it more. Most of the providers did not remember talking about the website with parents.

There were barriers to families using the PN materials. In pre- and post-surveys, weather was the most-commonly reported barrier to children’s PA, followed by time. In interviews, parents raised concerns that the nature toys and activity ideas were practical only for warm, dry weather. Parents wished that providers spent time discussing strategies for PA during cold and wet seasons. Parents pointed out that feeling tired, being busy with work and lack of time were also barriers to consistently supporting their children’s PA and time in nature.

Providers had mixed perceptions about the amount of time they had for PN during well-child visits; some felt the amount of time was sufficient, while others did not. Within the study context, providers found that not giving the intervention to all the patients seen each day led to forgetting to do the intervention with some of the participants that were enrolled in the study.

The cost of toys averaged $5.65 each (range $0.56–$12.50).

### Preliminary efficacy

There were no statistically significant changes in PA and outdoor time pre- and post-intervention (Table 8).

**Table 8:** Physical activity and outdoor time pre-and-post intervention

Question	Pre-intervention, *N* = 22	Post-intervention, *N* = 22	*p*-value
During the past week, on how many days did your child exercise, play a sport, or participate in physical activity for at least 20 min that made them sweat and breathe hard?	4.91 (2.04)	4.86 (1.52)	0.9
During the past week, on how many days did you exercise, play a sport, or participate in physical activity for at least 20 min that made you sweat and breathe hard?	3.14 (1.70)	3.59 (1.62)	0.14
During the past 7 days, on how many days were you and your child physically active TOGETHER for at least 20 minutes?	2.59 (2.04)	2.91 (1.87)	0.3
During the past 7 days, on how many days did you go outside with your child for a walk or play near your home or in a park?	3.18 (1.89)	3.55 (2.06)	0.3

## Discussion

This study demonstrates how we successfully adapted PN for a diverse group of families with children aged 4–10 years old, through an iterative process of engaging stakeholders and implementing feedback. PN was welcomed by providers and families and may be a practical intervention to promote outdoor active play during well-child visits. Pediatricians and other pediatric clinicians are uniquely positioned to encourage and support families in physical activity and nature contact but face some challenges in doing so. Building from these results, PN should be revised to better support active outdoor play during suboptimal weather and evaluated to test its efficacy in a fully-powered trial.

The nature toy was the most memorable aspect of the intervention for both providers and families and seemed to be a critical component of the intervention that may be worth the additional cost, time and storage space required by clinics. The toy facilitated activity that a parent and child could do *together*, in contrast to a parent watching their child play outdoors. While the current average cost of the nature toy is too high for scalability and potentially limits sustainability of the intervention, it is likely that the cost per toy would decrease when ordered in bulk. In primary care-based interventions that employ a toy, it will be key to balance the durability and quality of the toys with the cost, versatility, and age-appropriateness. Future cost-effectiveness studies may be warranted.

In contrast to the toy, there were mixed results about the brochure and most parents were not even aware that a website existed. Many parents desired more information about other outdoor activities, local resources including parks, and ways to be active during cold and wet times of the year. While the brochure and website contained some of this information, families were not aware of it so providers may need to more intentionally reference those resources in their discussions with families. In particular, the website could be a platform that can be customized with local resources (i.e. park finder by zip code, links to upcoming events, places to get outdoor gear, etc.) and kept updated, which could be useful for scaling this intervention. The fact that parents did not remember using the brochure or website supports existing literature stating that knowledge provision alone does not reliably result in behavior change.

Some providers felt there was not enough time to adequately explain PN within the allotted well-child visit. We suspect that work-flow fluidity would improve in a non-research context or with a different study design where the intervention could be employed during all well-checks. Moreover, parents wished that providers had spent more time directly motivating their children to be active and spend time in nature – especially during cold and wet times of the year. This has implications for how pediatric providers manage their time counseling during well-child visits and how other clinic staff may be able to support providers. Training providers to use PN materials by applying motivational interviewing strategies may help them incorporate PN more efficiently into their anticipatory guidance. It will also be important to consider how to help support parents to motivate their children to be active at home, such as through family-based activities. Positive reinforcement, reminders, and limiting sedentary behaviors and media use could be helpful strategies. Future studies could strive to learn from families that have successfully implemented behavioral changes.

There is a need for an adequately powered study to understand the impact of this intervention on behavioral outcomes including child PA as well as parent and child co-participation in physical activity. It will be important to evaluate how the intervention addresses barriers in larger studies – including the most-commonly cited barriers of weather and lack of time. Examining the core components of PN independently, and including provider counseling as an independent component of the intervention, will be helpful in prioritizing and allocating resources. We acknowledge that there is likely a synergistic effect of all the core intervention components, and that some may work better for individual families than others.

Limitations of our study include having a small sample of participants from one geographic area. This intervention was specifically adapted to address the barriers and meet the needs of the population of children who receive care at a single children’s clinic in Seattle. While this clinic serves an urban, racially and ethnically diverse, lower income population, the families participating may not be representative of those served by other clinics. We had difficulty recruiting families in the study because many did not answer their phone before their appointment or did not want to participate in research, which may have contributed to selection bias. While this version of PN has only undergone rigorous usability testing in English, next steps include adapting and testing the intervention to other languages to reach a broader population. Finally, evaluation of the efficacy of this intervention would need to include more rigorous and objective measures of outcomes such as PA.

Overall, our findings lay the foundation for future studies evaluating the efficacy of the PN intervention, as an evidence-based strategy to decrease disparities in children’s active play in nature. Ultimately, eliminating structural barriers to nature access through policy change and community investment will be necessary to improve equitable access to play and nature. We urge policy makers to implement plans to increase green space, fund improving the quality of green spaces that already exists in low-income communities, and consider less resource-intensive strategies such as adding gardens in childcare, school and community spaces [3]. In the meantime, pediatric providers can play a pivotal role in encouraging and promoting outdoor play through individual encounters with children and families.

### Supplementary Information

** Additional file 1: Supplementary file 1.** Adaptation Needs Assessment (Phase 1) interview script for parent/guardians.

** Additional file 2: Supplementary file 2.** Adaptation Needs Assessment (Phase 1) provider focus group guide.

** Additional file 3: Supplementary file 3.** Usability Testing (Phase 2) interview script for parent/guardians and clinic staff.

** Additional file 4: Supplemental file 4.** Pilot Evaluation (Phase 3) survey content.

** Additional file 5: Supplementary file 5.** Pilot Evaluation (Phase 3) interview script for parent/guardians.

** Additional file 6: Supplementary file 6.** Pilot Evaluation (Phase 3) interview script for providers.

## Data Availability

Interview transcripts analyzed during the current study are not publicly available but are available from the corresponding author on a reasonable request. All other data analyzed during this study are included in the published article.

## Abbreviations

PN: Project nature
PA: Physical activity
MVPA: Moderate-to-vigorous physical activity
