# Supplementary material for: Project nature: promoting outdoor physical activity in children via primary care
Source: BMC Prim Care. 2024 Feb 23;25:68. doi: 10.1186/s12875-024-02297-5 (PMC10885514; doi:10.1186/s12875-024-02297-5)
Supplement: Supplementary file 1 — Additional file 1: Supplementary file 1. Adaptation Needs Assessment (Phase 1) interview script for parent/guardians. [file 12875_2024_2297_MOESM1_ESM.docx]

**Supplementary file 1. Adaptation Needs Assessment (Phase 1) interview script for parent/guardians**

**ENGLISH**

**Introduction**:

Introductions. Reminder about purpose of the study. Details about the interview logistics

and audio recording. Opportunity for any questions. Obtain consent.

**Rapport questions:**

1. Would you please start by telling me the ages of any children you have living in your home?
2. I know that being a parent and keeping kids entertained during this pandemic has been hard for a lot of families. What are some things you have noticed your children enjoyed doing this fall?

**Outdoor active play:**

For our discussion today, I want you to think primarily about your children who are between the ages of 3 and 10. I believe you mentioned you have XX (#) children in that age range living with you. Is that correct?

1. Thinking back to **before the Covid virus upended our lives**, what are some ways your child enjoyed being physically active?

[PROBE: by this we mean doing things that cause them to breathe hard and raise their heart rate—moving their bodies as opposed to doing activities while sitting in a chair or laying down.]

1. What kinds of things got in the way of your child being physically active?

[PROBES: space, time, weather, don’t like it, don’t know what to do, competing priorities, don’t have the right equipment]. Anything else?

1. Still thinking about before Covid, can you give me some examples of activities your child did when playing outdoors?
2. Who did they do those with?
3. What kinds of things got in the way of your child playing outdoors?

[PROBES: safe places to go, time, weather, don’t like it, don’t know what to do, don’t have the right equipment, hard to get there]

1. Now I want to talk about nature. When I say, “being in nature,” what comes to mind for you?
2. Before Covid, how, if at all, did your child play in nature?

[PROBE: If doesn’t mention things close to home, ask, what about playing in nature close to home?]

1. Who did they do those things with?
2. Before Covid, what kinds of things got in the way of your child playing in nature?
3. There are many ways that a parent can care for a child and support them in growing up healthy and safe. Where does outdoor active play fit into the list of things that you think about as a parent?
4. Thinking about your child’s yearly well-child check-ups, did your child’s doctor or health care provider ever encourage you to take your child outside to play?
   1. [IF YES] When do you remember that conversation happening?
   2. [IF YES] What do you remember from that conversation?

**Impact of the Covid-19 pandemic [Note: these questions will only be asked during 1:1 interviews, not focus groups]**

Now I want you to think about how things have changed for your child since the Covid-19 pandemic started.

1. What are some ways your child enjoys being physically active now?
2. What kinds of things are getting in the way of your child being physically active?

[PROBES: Space, time, weather, don’t like it, don’t know what to do, competing priorities, don’t have the right equipment]

1. Can you give me some examples of activities your child does playing outdoors now?
2. Who do they do those with?
3. What kinds of things are getting in the way of your child playing outdoors?

[PROBES: safe places to go, time, weather, don’t like it, don’t know what to do, don’t have the right equipment, hard to get there]

1. We talked about your child playing in nature before Covid; I want you to think about now, during Covid. How, if at all, does your child play in nature now?

[If doesn’t mention things close to home] What about playing in nature close to home?

1. Who do they do those things with?
2. What kinds of things get in the way of your child playing in nature now?

**Project Nature Content**

We are going share some play kit materials we have previously made for younger children (ages 1-3) to encourage them to spend time outdoors, in nature. For each age, the child also gets a small toy to encourage outdoor play (share examples). We are thinking about best ways to adapt this for older children.

1. Do you think a play kit like this would be useful for a family with a child in {fill in their child’s age group: 3-5, 6-8, 9-10} age group?
   1. [IF NO] Why not?
   2. [IF YES] Which parts do you think would be useful to you for your child and family?
2. Which parts might not be useful for your child and your family?
3. What information would be important to include in the written materials?
4. Is there information specific to the Covid-19 pandemic that would be useful to include?
5. We know that parents have a lot to balance and many priorities in caring for their children—especially during this pandemic.
6. Do you think having a play kit like this would help your child spend more time outdoors and be more physically active? Why or why not?
7. What might get in the way of using the play kit?

[PROBES: Safety, space, weather, time, health concerns, etc.]

1. If you were the one to design a play kit that your child could use outdoors, what would it include?

[If they didn’t mention something to play with outside] Would you include something to play with outside? If so, what?

**Demographics**:

These last questions ask about you.

1. What is your child's current age?
2. What is your age?
3. What is your gender?
4. Are you of Mexican, Hispanic, or Latin American descent?
5. What is your race (check all that apply: African American or Black, American Indian or Alaska Native, Asian-American, Caucasian or white, Native Hawaiian or Pacific Islander, Other)
6. What languages are mostly spoken in your home?
7. Do you have an area where your child can play outside near your home – where your child can be noisy, run around and it is safe? If yes, what is that place?
8. Is there a park within 10-minute walk of your house that you feel your child is safe going to?
9. Is there a school within a 10 min walk of your house? If yes, is it open for you to use even while schools have been closed and/or after school hours?

**Wrap-up questions**:

1. We’ve reached the end of our interview. Is there anything I didn’t ask about that you’d like to tell me?

**SPANISH**

**Comunicación**:

1. ¿Podría comenzar informándome de las edades de los niños que viven en su hogar?

**Recreación activa al aire libre**:

Para nuestro debate del día de hoy, quiero que piense, en primer lugar, en los niños que tienen entre 3 y 10 años. Creo que mencionó que tiene XX (#) niños en ese rango de edad que viven con usted. ¿Es correcto?

Si nos remontamos al momento anterior a que la COVID pusiera en pausa nuestra vida…

**[Antes: físicamente activos]**

1. ¿Cuáles son algunas de las formas en las que su niño disfrutaba de mantenerse físicamente activo? [Indague: con esto, nos referimos a actividades que los hicieran respirar con mayor intensidad y que aumentaran su frecuencia cardíaca (mover su cuerpo en lugar de realizar actividades mientras están sentados o acostados)].
2. ¿Qué aspectos le impedían a su niño estar físicamente activo? [Indague: espacio, tiempo, condiciones del tiempo, no encontrar actividades que le gusten, no saber qué hacer, prioridades enfrentadas, falta del equipo adecuado]. ¿Algo más? [Antes: recreación al aire libre]
3. Aun pensando en el momento anterior a la COVID, ¿puede darme algunos ejemplos de actividades que su niño realizaba cuando jugaba al aire libre?
4. ¿Con quién las realizaba?
5. ¿Qué aspectos le impedían a su niño jugar al aire libre? [Indague: lugares seguros a los que ir, tiempo, condiciones del tiempo, no encontrar actividades que le gusten, no saber qué hacer, falta del equipo adecuado, dificultad para ir al aire libre].

**[Antes: estar en contacto con la naturaleza]**

1. Ahora me gustaría hablar de la naturaleza. Cuando digo “estar en la naturaleza”, ¿en qué piensa?
2. **Antes de la COVID**, ¿qué aspectos impedían el contacto de su niño con la naturaleza? [Si no se mencionan actividades realizadas cerca de casa, pregunte: ¿qué sucede con realizar actividades que permitan estar en contacto con la naturaleza cerca de casa?].
3. ¿Con quién realizaban esas actividades?
4. **Antes de la COVID**, ¿qué aspectos impedían el contacto de su niño con la naturaleza?
5. Si piensa en las revisiones médicas de atención preventiva infantil anuales de su niño, ¿el médico o proveedor de atención médica de su niño fomentaba la recreación al aire libre?
   1. [Si la respuesta es afirmativa] ¿Cuándo recuerda haber tenido esa conversación?
   2. [Si la respuesta es afirmativa] ¿Qué recuerda de esa conversación?

**Impacto de la pandemia de COVID-19 [Nota: estas preguntas solo se realizarán en la entrevista uno a uno, no en los grupos de discusión]**

Ahora quiero que piense en cómo han cambiado las cosas para su niño o sus niños de entre

3 y 10 años desde que comenzó la pandemia de COVID-19.

**[COVID-19: físicamente activos]**

1. ¿Cuáles son algunas formas en las que su niño disfruta de estar físicamente activo actualmente?
2. ¿Qué aspectos impiden que su niño esté físicamente activo? [Indague: espacio, tiempo, condiciones del tiempo, no encontrar actividades que le gusten, no saber qué hacer, prioridades enfrentadas, falta del equipo adecuado].

**[COVID-19: recreación al aire libre]**

1. ¿Puede darme algunos ejemplos de actividades que su niño realiza al aire libre actualmente?
2. ¿Con quién realiza esas actividades?
3. ¿Qué aspectos impiden que su niño realice actividades al aire libre? [Indague: lugares seguros a los que ir, tiempo, condiciones del tiempo, no encontrar actividades que le gusten, no saber qué hacer, falta del equipo adecuado, dificultad para ir al aire libre].

**[COVID-19: estar en contacto con la naturaleza]**

1. Hablamos del contacto de su niño con la naturaleza antes de la COVID. Quiero saber qué piensa ahora, durante la COVID. ¿Cómo entra su niño en contacto con la naturaleza ahora, si es que lo hace? [Si no se mencionan actividades realizadas cerca de casa, pregunte qué sucede con realizar actividades que permitan entrar en contacto con la naturaleza cerca de casa].
2. ¿Con quién realizan esas actividades?
3. ¿Qué aspectos impiden que su niño entre en contacto con la naturaleza ahora?

**Contenido del proyecto Naturaleza**

Compartiremos algunos materiales de juego que hemos realizado previamente para niños más jóvenes (de entre 1 y 3 años) con el fin de fomentar que pasen tiempo al aire libre, en contacto con la naturaleza. Para cada edad, el niño también recibe un pequeño juguete para fomentar el juego al aire libre (compartir ejemplos). Pensamos en las mejores formas de adaptar esto a niños más grandes.

1. ¿Cree que los materiales de juego como estos podrían ser útiles para una familia con un niño de [completar con el grupo etario del niño: 3 a 5 años, 6 a 8 años, 9 a 10 años]?
   1. [Si la respuesta es negativa] ¿Por qué no?
   2. [Si la respuesta es afirmativa] ¿Qué partes de estos materiales usted cree que serían útiles para usted, su niño y su familia?
2. ¿Qué partes pueden no ser útiles para su niño y su familia?
3. ¿Qué información sería importante incluir en los materiales escritos?
4. ¿Hay información específica sobe la pandemia de COVID-19 que sería útil incluir?

Sabemos que los padres y las madres deben encontrar el equilibrio entre muchos aspectos y establecer prioridades en cuanto a la atención de sus hijos, en especial durante esta pandemia.

1. ¿Cree que contar con materiales de juego como estos ayudaría a su niño a pasar más tiempo al aire libre y estar físicamente más activo? ¿Por qué o por qué no?
2. ¿Qué cree que podría impedir el uso de estos materiales de juego? (por ejemplo, seguridad, espacio, condiciones del tiempo, tiempo, inquietudes de salud, etc.)
3. Si fuese usted quien diseñara materiales de juego para que su niño utilice al aire libre, ¿qué incluiría?
   1. [Si no se menciona nada para jugar al aire libre] ¿Incluiría algo para jugar al aire libre? Si es así, ¿qué?

**Final de las preguntas**:

1. Hemos llegado al final de nuestra entrevista. ¿Hay algo que no le haya preguntado que le gustaría contarme?
