# Supplementary material for: Project nature: promoting outdoor physical activity in children via primary care
Source: BMC Prim Care. 2024 Feb 23;25:68. doi: 10.1186/s12875-024-02297-5 (PMC10885514; doi:10.1186/s12875-024-02297-5)
Supplement: Supplementary file 2 — Additional file 2: Supplementary file 2. Adaptation Needs Assessment (Phase 1) provider focus group guide. [file 12875_2024_2297_MOESM2_ESM.docx]

**Supplementary file 2. Adaptation Needs Assessment (Phase 1) provider focus group guide**

**Introduction**:

Introductions. Reminder about purpose of the study. Details about the focus group logistics

and audio recording. Opportunity for any questions. Obtain consent.

**Rapport:**

Let’s start by going around and each person telling where you practice, your specialty and the patient population you serve. Because we are not sitting around a table, I’ll call on each of you.

For our discussion today, I want you to think primarily about the families you serve who are struggling financially, have fewer resources, or are on Washington Apple Health. Specifically, we’re interested in families with children between the ages of 3 and 10 who come in for well-child visits.

**Outdoor active play:**

We are interested in your feedback on how health care providers can best inform these families with preschool and elementary school aged children about how to encourage outdoor active play, all the time, but particularly during the current Covid-19 pandemic.

1. Thinking first about families with children 3-5, how, if at all, has the topic of outdoor active play during Covid-19 come up between you and your patients?
2. When you think about all of the topics you could cover with parents during a well-child visit for children 3-5, where does providing guidance about outdoor active play fall in those priorities if at all? How important is this to you as a provider?
3. Now thinking about an actual day in the clinic, how often do you actually provide guidance about outdoor active play to families with children 3-5?
4. What gets in the way?
5. Do you have any resources to provide parents about outdoor play? If so, what? (probes: after visit summaries, handouts, brochures, booklets, etc.)
6. Thinking about resources you have or you’d like to have, what format would you like this information to be in? (some options: handout, link to website, fridge magnet, book for child)
7. Now I want you to think about families with children ages 6-10, how, if at all, has the topic of outdoor active play during Covid-19 come up between you and these patients?
8. When you think about all of the topics you could cover with parents during a well-child visit for children 6-10, where does providing guidance about outdoor active play fall in those priorities if at all? How important is this to you as a provider?
9. Now thinking about an actual day in the clinic, how often do you actually provide guidance about outdoor active play to families with children 6-10?
10. What gets in the way?
11. Do you have any resources to provide parents about outdoor play? If so, what?

[PROBES: after visit summaries, handouts, brochures, booklets, etc.]

1. Thinking about resources you have or you’d like to have, what format would you like this information to be in? (some options: handout, link to website, fridge magnet, book for child)

**Project Nature Content**:

Now let’s shift to talking about actual materials that could be used to promote outdoor play for your patients.

First, I want to share some materials for a play kit made for younger children (ages 1-3) that have already been developed. The goal of the play kit is to encourage families to take their small children outdoors to play. At each well-child visit, parent gets a booklet/brochure and the child gets an age-appropriate small item to encourage outdoor play (share examples).

This example is for 1-3 year olds, now imagine a similar, age-appropriate play kit for your low-resource families with 3-10 year olds.

1. Thinking back to my earlier question about how you prioritize discussing outdoor play, would having this kind of play kit to share with families make it easier for you to provide guidance about outdoor play during a well-child visit? Why or why not?
2. Do you think an age-appropriate play kit like this would motivate your patients and their families to spend more time in outdoor play? Why or why not? What would get in the way? (some options: safety, space, weather, time, health concerns, etc.)
3. What are some challenges that might prevent your clinic from providing play kits to families?

**Wrap up questions**:

1. We’ve reached the end of our focus group. Is there anything I didn’t ask about that you’d like to tell me?

**Demographics**: Questions to be asked during scheduling call, after obtaining consent

1. What is your age?
2. What is your gender?
3. Are you of Mexican, Hispanic, or Latin American descent?
4. What is your race? (check all that apply: African American or Black, American Indian or Alaska Native, Asian-American, Caucasian or white, Native Hawaiian or Pacific Islander, Other)
5. How many years have you been practicing pediatrics?
