# Supplementary material for: Project nature: promoting outdoor physical activity in children via primary care
Source: BMC Prim Care. 2024 Feb 23;25:68. doi: 10.1186/s12875-024-02297-5 (PMC10885514; doi:10.1186/s12875-024-02297-5)
Supplement: Supplementary file 3 — Additional file 3: Supplementary file 3. Usability Testing (Phase 2) interview script for parent/guardians and clinic staff. [file 12875_2024_2297_MOESM3_ESM.docx]

**Supplementary file 3. Usability Testing (Phase 2) interview script for parent/guardians and clinic staff**

**ENGLISH**

**Qualitative questions**:

1. What do you like best about Project Nature? Why?
2. What do you like least about Project Nature? Why?
3. What would you change about the Project Nature toy?
4. What would you change about the Project Nature handout?
5. Would your family use a Project Nature kit if your child was given one? Why or why not?
6. Anything else?

**Usability questions (from Lyon et al.’s Usability Evaluation for Evidence-Based Psychosocial Interventions Scale, 2020)**

1. I would like to use Project Nature often.

| 1 | 2 | 3 | 4 | 5 |
| --- | --- | --- | --- | --- |
| Strongly disagree | Disagree | Neither disagree nor agree | Agree | Strongly agree |

1. Project Nature was hard to understand.

| 1 | 2 | 3 | 4 | 5 |
| --- | --- | --- | --- | --- |
| Strongly disagree | Disagree | Neither disagree nor agree | Agree | Strongly agree |

1. Project Nature would be easy to use.

| 1 | 2 | 3 | 4 | 5 |
| --- | --- | --- | --- | --- |
| Strongly disagree | Disagree | Neither disagree nor agree | Agree | Strongly agree |

1. I would need help to use Project Nature.

| 1 | 2 | 3 | 4 | 5 |
| --- | --- | --- | --- | --- |
| Strongly disagree | Disagree | Neither disagree nor agree | Agree | Strongly agree |

1. Most people could learn to use Project Nature very quickly.

| 1 | 2 | 3 | 4 | 5 |
| --- | --- | --- | --- | --- |
| Strongly disagree | Disagree | Neither disagree nor agree | Agree | Strongly agree |

1. Project Nature would be very hard to use.

| 1 | 2 | 3 | 4 | 5 |
| --- | --- | --- | --- | --- |
| Strongly disagree | Disagree | Neither disagree nor agree | Agree | Strongly agree |

1. I would feel confident using Project Nature

| 1 | 2 | 3 | 4 | 5 |
| --- | --- | --- | --- | --- |
| Strongly disagree | Disagree | Neither disagree nor agree | Agree | Strongly agree |

1. I would need to learn a lot of things before I could get going with Project Nature

| 1 | 2 | 3 | 4 | 5 |
| --- | --- | --- | --- | --- |
| Strongly disagree | Disagree | Neither disagree nor agree | Agree | Strongly agree |

**Demographic questions – for parents/guardians**:

1. What is your gender? (male, female, non-binary, prefer to not answer)
2. How old are you?
3. How many children do you have age 3-12?
4. How old is your child (who is attending an appointment today)? (if you have more than 1 child between the age of 3-10, please think about the one whose birthday is coming up next)
5. What is your child’s gender? (male, female, non-binary, prefer to not answer)
6. Which of the following best describes you? (Asian or Pacific Islander, Black or African American, Hispanic or Latino, Native American or Alaskan Native, White or Caucasian, Multiracial or Biracial, a race/ethnicity not listed here)
7. Are you of Mexican, Hispanic, or Latin American descent?

**Demographic questions – for clinic staff**:

1. What is your gender? (male, female, non-binary, prefer to not answer)
2. How old are you?
3. Which of the following best describes you? (Asian or Pacific Islander, Black or African American, Hispanic or Latino, Native American or Alaskan Native, White or Caucasian, Multiracial or Biracial, a race/ethnicity not listed here)
4. Are you of Mexican, Hispanic, or Latin American descent?
5. What is your role at OBCC?
6. How many years have you worked in healthcare since finishing your training?

**SPANISH**

**Guía de entrevistas cualitativas**:

1. ¿Qué es lo que más le gusta del Proyecto Naturaleza? ¿Por qué?
2. ¿Qué es lo que menos le gusta del Proyecto Naturaleza? ¿Por qué?
3. ¿Qué cambiaría del juguete del Proyecto Naturaleza?
4. ¿Qué cambiaría del folleto del Proyecto Naturaleza?
5. ¿Utilizaría su familia un kit del Proyecto Naturaleza si se le diera uno a su hijo? ¿Por qué o por qué no?
6. ¿Algo más?

**Funcionalidad**:

1. Me gustaría utilizar el Proyecto Naturaleza con frecuencia.

| 1 | 2 | 3 | 4 | 5 |
| --- | --- | --- | --- | --- |
| Totalmente en  desacuerdo | En desacuerdo | Ni en desacuerdo  ni de acuerdo | De acuerdo | Totalmente  de acuerdo |

1. El Proyecto Naturaleza fue difícil de entender.

| 1 | 2 | 3 | 4 | 5 |
| --- | --- | --- | --- | --- |
| Totalmente en  desacuerdo | En desacuerdo | Ni en desacuerdo  ni de acuerdo | De acuerdo | Totalmente  de acuerdo |

1. El Proyecto Naturaleza sería fácil de usar.

| 1 | 2 | 3 | 4 | 5 |
| --- | --- | --- | --- | --- |
| Totalmente en  desacuerdo | En desacuerdo | Ni en desacuerdo  ni de acuerdo | De acuerdo | Totalmente  de acuerdo |

1. Necesitaría ayuda para usar el Proyecto Naturaleza.

| 1 | 2 | 3 | 4 | 5 |
| --- | --- | --- | --- | --- |
| Totalmente en  desacuerdo | En desacuerdo | Ni en desacuerdo  ni de acuerdo | De acuerdo | Totalmente  de acuerdo |

1. La mayor parte de las personas podría aprender a usar el Proyecto Naturaleza muy rápidamente.

| 1 | 2 | 3 | 4 | 5 |
| --- | --- | --- | --- | --- |
| Totalmente en  desacuerdo | En desacuerdo | Ni en desacuerdo  ni de acuerdo | De acuerdo | Totalmente  de acuerdo |

1. El Proyecto Naturaleza sería muy difícil de usar.

| 1 | 2 | 3 | 4 | 5 |
| --- | --- | --- | --- | --- |
| Totalmente en  desacuerdo | En desacuerdo | Ni en desacuerdo  ni de acuerdo | De acuerdo | Totalmente  de acuerdo |

1. Me sentiría seguro usando el Proyecto Naturaleza.

| 1 | 2 | 3 | 4 | 5 |
| --- | --- | --- | --- | --- |
| Totalmente en  desacuerdo | En desacuerdo | Ni en desacuerdo  ni de acuerdo | De acuerdo | Totalmente  de acuerdo |

1. Tendría que aprender muchas cosas antes de empezar con el Proyecto Naturaleza.

| 1 | 2 | 3 | 4 | 5 |
| --- | --- | --- | --- | --- |
| Totalmente en  desacuerdo | En desacuerdo | Ni en desacuerdo  ni de acuerdo | De acuerdo | Totalmente  de acuerdo |

**Preguntas demográficas – para padres/tutores**:

1. ¿Cuál es su género? (Hombre, mujer, no binario, prefiero no responder)
2. ¿Qué edad tiene?
3. ¿Cuántos hijos tiene de 3 a 12 años de edad?
4. ¿Cuántos años tiene su hijo (el que asiste a la cita de hoy)? (Si tiene más de 1 hijo entre 3 y 10 años de edad, piense en el niño cuya fecha de cumpleaños es más cercana.)
5. ¿Cuál es el género de su hijo? (Hombre, mujer, no binario, prefiero no responder)
6. ¿Cuál de las siguientes opciones lo describe mejor? (Asiático o Nativo de las Islas del Pacífico, Negro o Afroamericano, Hispano o Latino, Nativo Americano o Nativo de Alaska, Blanco/Caucásico, Multirracial o Birracial, Raza/origen étnico no listado aquí)
7. ¿Es usted de ascendencia mexicana, hispana o latinoamericana?
