# Supplementary material for: Project nature: promoting outdoor physical activity in children via primary care
Source: BMC Prim Care. 2024 Feb 23;25:68. doi: 10.1186/s12875-024-02297-5 (PMC10885514; doi:10.1186/s12875-024-02297-5)
Supplement: Supplementary file 4 — Additional file 4: Supplemental file 4. Pilot Evaluation (Phase 3) survey content. [file 12875_2024_2297_MOESM4_ESM.docx]

**Supplemental file 4. Pilot Evaluation (Phase 3) Survey Content**

**Notes:** Surveys were be formatted for online and paper distribution depending on school preference following best practices for survey formatting. Additionally, instructions were added and/or clarified (e.g. change “place a check in the circle” to “click your answer” for online surveys)]. All questions are for pre and post unless stated otherwise in column 1.

| **Domain** | **Questions** |
| --- | --- |
| Parent and child demographic characteristics **(PRE-Survey only)** | What is your gender identity? *[Response options: man, woman, trans man/transmasculine, trans woman/transfeminine, nonbinary or genderqueer, identity not listed (please specify:____), I choose not to answer this question]*  What was the sex recorded on your original birth certificate? *[Response options: Male, female, I choose not to answer this question]*    Are you Hispanic, Latino, or of Spanish origin? *[Response options: Yes, No, I choose not to answer this question]*    Which of the following best describes your racial background? (Choose all that apply) *[Response options: Asian, Black or African American, Middle Eastern or North African, Multiracial or mixed race, Native American and/or Alaska Native, Native Hawaiian and/or Pacific Islander, White, None of the above or choose to self-describe: _______, I choose not to answer this question]*  How old are you? *[Response options: ____ years]*  How many children do you have between the ages of 4-12? *[Response options: 0-6+]*  How old is your child (who is attending their Well Child Checkup)? *[Response options: 4-10 years-old]*  *If you have more than 1 child between the age of 3-12, please think about the one whose birthday is coming up next. When questions in this survey ask about “your child” think about* ***this*** *child.*  What is your child’s gender identity? *[Response options: boy, girl, nonbinary or genderqueer, questioning or exploring identity, Identity not listed (please specify:______), I choose not to answer this question]*  What is the sex of your child recorded on their birth certificate? *[Response options: Male, female, I choose not to answer]*  Is your child Hispanic, Latino, or of Spanish origin? *[Response options: Yes, No, I choose not to answer this question]*    Which of the following best describes your child’s racial background? (Choose all that apply) *[Response options: Asian, Black or African American, Middle Eastern or North African, Multiracial or mixed race, Native American and/or Alaska Native, Native Hawaiian and/or Pacific Islander, White, None of the above or choose to self-describe: _______, I choose not to answer this question]* |
| Parent report of child health diagnosis  **(PRE-Survey only)** | Has your child ever been diagnosed with or in the process of being evaluated for any of the following: *[Response options: yes, no, prefer not to respond]*  Chronic mental health condition (i.e. anxiety, depression)  Behavioral / neurodevelopmental condition (i.e. ADHD, Autism, learning disability)  Chronic physical health condition (i.e. asthma, diabetes, inflammatory bowel disease) |

| Parent report of child physical activity | During the past week, on how many days did **your child** exercise, play a sport, or participate in physical activity **for at least 20 min** that made (him/her) sweat and breathe hard? *[Response options: 0 days, 1 day, 2 days, 3 days, 4 days, 5 days, 6 days, 7 days]*  During the past week, on how many days did **you** exercise, play a sport, or participate in physical activity **for at least 20 min** that made you sweat and breathe hard? *[Response options: 0 days, 1 day, 2 days, 3 days, 4 days, 5 days, 6 days, 7 days]*  During the past 7 days, on how many days were **you and your child** physically active **TOGETHER** for at least **20 minutes**? Add up all the time you spent in any kind of physical activity that increased your heart rate and made you breathe hard some of the time). *[Response options: 0 days, 1 day, 2 days, 3 days, 4 days, 5 days, 6 days, 7 days]* |
| --- | --- |
| Outdoor time with child | In the past week, on how many days did you go outside with your child for a walk or to play near your home or in a park? [*Response options: numeric response from 0-7]* |
| Outdoor Recreation – Access | Do you have a park that you can safely walk to within 10 minutes from your home? *[Response options: yes, no, I don’t know]* |
| Nature Connectedness Measure  (From: Richardson et al. “A Measure of Nature Connectedness for Children and Adults: Validation, Performance, and Insights.” *Sustainability*, 2019, 11(12), 3250, https://doi.org/10.3390/su11123250) | *[Response options for the following: 7 points “completely disagree” (1) to “completely agree” (7)]*   - I always find beauty in nature - I always treat nature with respect - Being in nature makes me very happy - Spending time in nature is very important to me - I find being in nature really amazing - I feel part of nature - Being in nature makes my child very happy - My child finds being in nature really amazing - My child always find beauty in nature - Spending time in nature is very important to my child - My child always treat nature with respect - My child feels part of nature |
| Pediatric provider behaviors | In the past year, has your child’s medical provider discussed the following with you? *[Responses: yes/no]*   - Limiting screen time for your child - Encouraging your child to be physically active every day - Having a regular bedtime routine for your child - Encouraging your child to spend time outdoors every day - Limiting sugary drinks for your child |
| Perceived barriers of physical activity | Thinking about the past week, how much have each of the following gotten in the way of your child being physically active for at least 20 minutes/day, every day? *[Response options: never, sometimes, often, always]*   - They don’t like doing physical activity - They don’t know what to do - It’s not safe to go outside in my neighborhood to play/be active - There are no parks or greenspaces nearby - They don’t have anyone to play/be active with - The weather is not good for outdoor activities - We don’t have the right equipment to play/be active - We don’t have enough time - I’m worried they/we might get COVID-19 - We want to do online physical activity but we don’t have the right resources (e.g., laptop, wifi) |
| Readiness to change | *[Response options for the following questions: I am not interested in making changes at this time; I am not ready to make changes yet, but want to talk more; I am ready to make some changes now and would like help; I am already helping my child to be more active and don't feel there is much more to do]*  How do you feel about making some changes to help your child be more active?  How do you feel about making some changes to help your child get outdoors more often? |
| Perceived benefits of physical activity | How important is it to you that your child is physically active at least 20 minutes each day?  *[Response options: 7 points from “not at all important” to “extremely important”]* |
| Self-efficacy questions | How sure are you that you will do each of the following: *[Response options: 4=very sure, 3=pretty sure, 2=a little sure, 1=not sure at all]*  Support your child in being PA for at least 20 min /day, every day…   - When you are feeling tired - When it’s raining or cold outside - When you are feeling down or depressed - When you have too much work to do at home - When there are other more interesting things to do - When your family or friends do not provide any kind of support - When you don’t really feel like it - When your child doesn’t really feel like it |

**POST-Survey Only**

| Nature kit recall | What did your nature kit include? *[Response: check all that apply]*   - Information about activities my child can do in nature - A Book - Chalk - Jump rope - Water bottle - Bubble wand - Scavenger cards - Magnifying glass - Pocket kite - Frisbee - Seed packets and shovel - I don’t know/can’t remember |
| --- | --- |
| Nature kit – specifics | Did you use the kit? *[Responses: yes/no]*  How did you feel about the following items related to Project Nature? *[Responses: strongly liked, somewhat liked, neutral, somewhat disliked, strongly disliked, don’t remember getting this]*   - The information discussed by your pediatrician - The brochure given to you - The outdoor play item you received |
| Acceptability  (From: Proctor et al. “Outcomes for implementation research: Conceptual distinctions, measurement  challenges, and research agenda.” *Administration and Policy in Mental Health and Mental Health*  *Services Research*, 2011, 38, 65-76. doi: 10.1007/s10488-010-0319-7) | *[Responses for each of the following: completely disagree; disagree; neither agree nor disagree; agree; completely agree]*   - The Project Nature brochure and toy meet my approval. - The Project Nature brochure and toy are appealing to me. - I like the Project Nature brochure and toy. - I welcome The Project Nature brochure and toy. |
