# Supplementary material for: Project nature: promoting outdoor physical activity in children via primary care
Source: BMC Prim Care. 2024 Feb 23;25:68. doi: 10.1186/s12875-024-02297-5 (PMC10885514; doi:10.1186/s12875-024-02297-5)
Supplement: Supplementary file 5 — Additional file 5: Supplementary file 5. Pilot Evaluation (Phase 3) interview script for parent/guardians. [file 12875_2024_2297_MOESM5_ESM.docx]

**Supplementary file 5. Pilot Evaluation (Phase 3) interview script for parent/guardians**

**Introduction**:

Introduction. Opportunity for any questions. Obtain consent.

**Rapport**:

1. To start out, can you share with me some things your family enjoys doing together to have fun?

**Interaction with healthcare provider**:

For our interview today, I want you to think specifically about [child’s name] even if you have other children in your family.

1. Think back to [child’s name]’s most recent well-child checkup at the doctor’s office. About when was that last visit for [child’s name]?
2. At that visit, did your child’s doctor talk with you about physical activity or outdoor time?
   1. What do you remember from that conversation? [Probe: Is there anything else you remember?]
   2. Is there anything you wish the doctor had said about physical activity or outdoor time during the visit?

**Project Nature acceptability / usability**:

At that well-child visit for [child’s name] I believe you and [child’s name] were given a Project Nature brochure and toy.

1. Do you remember the toy that [child’s name] received during their doctor’s visit?
2. What did you think about this toy when [child’s name] chose it?
3. Did you or your family use the Project Nature brochure and the toy?

[IF NO]

- 1. That is ok. Not every family used it, but we would like to understand why. Do you mind sharing some reason why you and [child’s name] did not use it?

[Probe] Can you tell me more about this…

[IF YES]

- 1. About how many times did your family use it?
  2. Can you give me some examples of where and how your family used it….

[Probe] What are some things your family did with it?

1. What did your family like best about the Project Nature brochure and toy - why?
2. What was something about the nature Project Nature brochure and toy that did not work well for your family - why?
3. Did talking with Ada's doctor and getting the Project Nature brochure and toy help your family get outside?
   1. [IF YES] Could you describe how it helped you?
   2. [IF NO] Why do you think it didn't help?
   3. [NOTE: If they talk ONLY about the brochure, ASK about the toy, and vice versa]
4. What are some ways we could make the Project Nature brochure and toy better?

**Project Nature website acceptability / usability**:

1. Did you visit the website [www.projectnaturewa.com](http://www.projectnaturewa.com)?

[IF YES]

- 1. About how many times did you visit the website?
  2. Tell me about how you used it.
  3. What did you do on the website?
  4. What did your family like best about the website? Why?
  5. What was something about the website that did not work well for your family? Why?
  6. What are some ways we could make the website better?

[IF NO]

- 1. That is OK. Not, every family visited the website, and we would like to understand why. So, could you tell me some reasons you and Aya did not use it?

**Wrap up Questions**

We’ve reached the end of our interview. Is there anything I didn’t ask about that you’d like to

tell me? Do you have any questions for me?
