# Supplementary material for: Project nature: promoting outdoor physical activity in children via primary care
Source: BMC Prim Care. 2024 Feb 23;25:68. doi: 10.1186/s12875-024-02297-5 (PMC10885514; doi:10.1186/s12875-024-02297-5)
Supplement: Supplementary file 6 — Additional file 6: Supplementary file 6. Pilot Evaluation (Phase 3) interview script for providers. [file 12875_2024_2297_MOESM6_ESM.docx]

**Supplementary file 6. Pilot Evaluation (Phase 3) interview script for providers**

**Introduction**:

Introduction. Opportunity for any questions. Obtain consent.

**Project Nature Feasibility**:

1. According to our reports, X number of your patients participated in our Project Nature pilot. Does that sound about right?
2. What if anything do your remember about the Project Nature intervention?
3. How do you remember your patients responded to the project Nature toy, brochure or website?
4. What were the hardest things about doing the PN intervention in your clinic?

[OPTIONAL PROMPTS]:

- 1. Patient flow
  2. Multiple children present
  3. Toy storage
  4. Time crunch/Length of visit

1. After doing the Project Nature intervention, how feasible do you think it is to implement it as an ongoing practice in your clinic’s well-child visits?
